# The microbial tryptophan metabolite indole acts on the gastrointestinal tract to improve glucose homeostasis in a mouse model of diabetes by enhancing GLP-1 secretion and L cell differentiation

**DOI:** 10.1007/s00125-026-06688-4

**Published:** 2026-03-04

**Authors:** Phyllis Phuah, Mariana Norton, Sijing Cheng, Anna G. Roberts, Daniela Pirri, Leah Meyer, Pei-En Chung, Cecilia Dunsterville, Rafal Karwowski, Brian Y. H. Lam, Emile Otsubo, Sofia Aleksashina, Fiona M. Gribble, Frank Reimann, Aylin C. Hanyaloglu, Giles S. H. Yeo, Gavin A. Bewick, Ben Jones, Bryn Owen, Kevin G. Murphy

**Affiliations:** 1 Section of Endocrinology and Investigative Medicine, Division of Diabetes, Endocrinology and Metabolism, Department of Metabolism, Digestion and Reproduction, Faculty of Medicine, Imperial College London, Hammersmith Hospital, London, UK; 2 MRC Metabolic Diseases Unit, Institute of Metabolic Science Metabolic Research Laboratories, University of Cambridge, Cambridge, UK; 3 Institute of Reproductive and Developmental Biology, Department of Metabolism, Digestion and Reproduction, Imperial College London, London, UK; 4 Diabetes and Obesity Theme, School of Cardiovascular and Metabolic Medicine and Sciences, Faculty of Life Sciences and Medicine, King’s College London, London, UK

**Keywords:** Glucagon-like peptide-1, Glucose homeostasis, Transient receptor potential channel subtype A1

## Abstract

**Aims/hypothesis:**

Growing evidence implicates gut microbiota-derived metabolites in metabolic homeostasis. Indole, a microbial tryptophan metabolite, has been reported to enhance glucagon-like peptide-1 (GLP-1) secretion in vitro, and its derivatives have been inversely associated with risk of type 2 diabetes. We hypothesised that indole acts via the gastrointestinal tract to modulate glucose homeostasis, and tested this hypothesis using in vitro and in vivo models.

**Methods:**

We measured GLP-1 secretion from cultured murine enteroendocrine cells, and evaluated intraperitoneal glucose tolerance and hormone secretion in mice following indole treatment. Subsequently, the impact of indole on intestinal epithelial cell fate and L cell number was examined using murine ileal organoid cultures and in vivo. Finally, we explored the effect of chronic indole administration on metabolic outcomes in a murine model of type 2 diabetes.

**Results:**

Indole stimulated in vitro GLP-1 secretion in a concentration-dependent manner, and improved acute glucose management in vivo. Additionally, we demonstrate that indole drives enteroendocrine L cell differentiation in murine ileal organoids, resulting in increased L cell density and longer-term glucoregulatory benefits in vivo. Finally, sub-chronic indole administration improved glucose tolerance and insulin sensitivity in a diabetic mouse model.

**Conclusions/interpretation:**

Our findings identify indole as a glucose-lowering molecule that acts on the gut, and raise the possibility of incorporating indole into nutraceutical supplements to aid in the treatment or prevention of type 2 diabetes. This study highlights the importance of gut microbiota-derived metabolites in metabolic health and opens new avenues for developing novel strategies to combat type 2 diabetes.

**Data availability:**

RNA sequencing data are available from the Gene Expression Omnibus under accession number GSE306720.

**Graphical Abstract:**

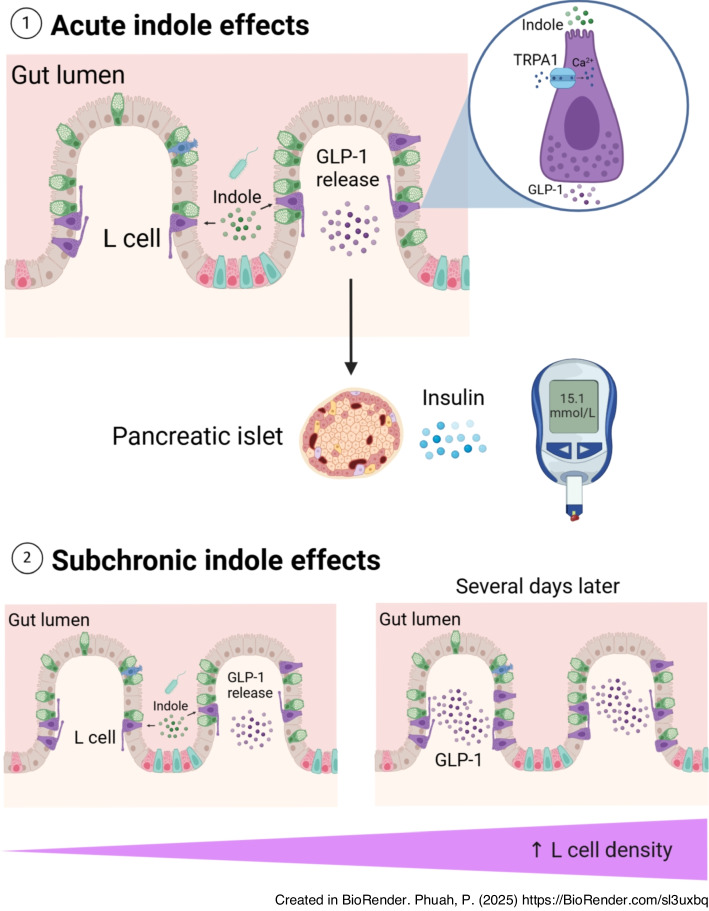


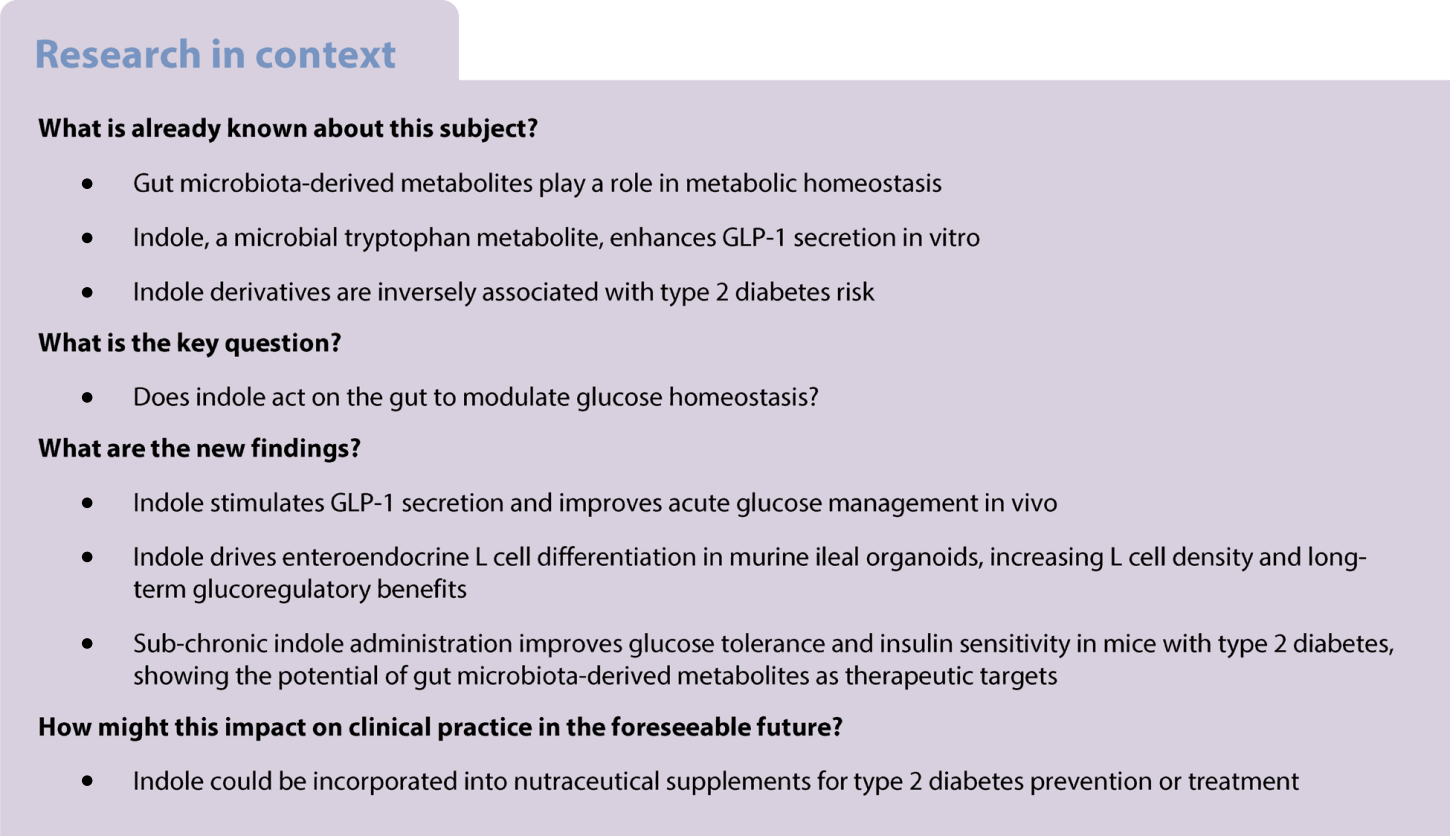


## Introduction

The gastrointestinal tract plays a crucial role in maintaining metabolic and glucose homeostasis. Beyond its role in digestion and absorption, it regulates systemic metabolism and glucose levels through modulation of gut hormone secretion and the gut microbiome [1, 2]. This intricate relationship between the gut, its microbial inhabitants, and host metabolism is essential for health, and has implications for metabolic disorders.

Gut microbial dysbiosis is implicated in the pathophysiology of metabolic disorders such as obesity and type 2 diabetes. Nutrient-derived metabolites are key mediators of host–microbiota crosstalk and hence metabolic homeostasis [3]. An inverse association between plasma concentrations of derivatives of indole, a bacterial catabolite of the essential amino acid l-tryptophan, and type 2 diabetes risk was previously identified [4]. While most dietary tryptophan is absorbed for protein synthesis or used as a substrate for the kynurenine and serotonin pathways, 4–6% is metabolised by colonic bacteria to generate indole and its derivatives. Indole is the most abundantly generated microbial tryptophan catabolite, with concentrations of 0.2–1 mmol/l detected in human and rodent faeces [5]. Locally, indole enhances intestinal epithelial integrity and attenuates inflammation [6, 7]. However, recent studies have also reported that indole modulates gut hormone release [8, 9]. Specifically, indole was found to stimulate secretion of glucagon-like peptide-1 (GLP-1) from GLUTag cells following short exposures in vitro [8, 9]. GLP-1 is an incretin that is secreted by enteroendocrine L cells. Current type 2 diabetes pharmacotherapies include synthetic GLP-1 analogues, although their utility can be limited by side effects. Accordingly, an alternative treatment strategy is to modulate endogenous GLP-1 release using dietary metabolites. We hypothesised that indole modulates glucose homeostasis by modulating GLP-1 signalling. The aims of this study were to: (1) assess the acute effects of indole on GLP-1 secretion and glucose metabolism; (2) examine the impact of indole on the GLP-1 system; and (3) evaluate the metabolic effects of chronic indole treatment.

## Methods

### Animals

All mice used were 8–12 weeks old and free of specific pathogens. They weighed 20–32 g, apart from TALLYHO mice, which weighed 30–40 g. Male C57Bl/6J mice (Charles River) were used unless otherwise stated. Mice on a 129svJ background were bred in-house. GluCreERT2 mice were generated by F. Gribble and F. Reimann, and crossed with Ai95(RCL-GCaMP6f)-D mice (The Jackson Laboratory, USA; RRID:IMSR_JAX:028865; https://www.jax.org/strain/028865). GLP-1 receptor (GLP-1R *fl/fl*) mice were generated by R. Seeley at the University of Michigan and crossed with Pdx1-Cre/Esr1 mice (The Jackson Laboratory, USA; RRID:IMSR_JAX:024968; https://www.jax.org/strain/024968) to generate Pdx1-CreERT; Glp1r *fl/fl* mice. Tamoxifen (100 mg/kg) was i.p. injected for 4 days to induce beta cell knockout of the GLP-1R. Both male and female GluCreERT2;Ai95-D and Pdx1-Cre;GLP-1R *fl/fl* mice were used. Only male TALLYHO/JnG mice (The Jackson Laboratory, USA; RRID:IMSR_JAX:005314; https://www.jax.org/strain/005314), purchased from the Jackson Laboratory, were used because only the male mice develop characteristics of diabetes, including glucose intolerance and insulin resistance. A total of 388 mice were used. Each experimental group consisted of a minimum of six mice. The numbers of animals/experiments/cells for each study are given in the results and figure legends. Group sizes were determined based on prior studies and on power calculations (where applicable) to ensure adequate statistical power.

The mice were housed in groups in individually ventilated cages containing standard bedding with a 12 h light/dark cycle (lights on at 07:00 hours, lights off at 19:00 hours) with ad libitum access to standard laboratory RM1 chow and water, and cardboard tubes for environmental enrichment. Procedures were carried out during the light cycle. All procedures were carried out according to the UK Animals (Scientific Procedure) Act 1986, and approved by the Imperial College London Animal Welfare and Ethical Review Body. Our study followed ARRIVE reporting guidelines. The manipulations used mice from different groups (randomised by body weight) in a mixed sequence to avoid confounders, and investigators were blinded regarding mouse genotype and treatment.

### Intraperitoneal glucose tolerance test

Glucose tolerance tests were performed on mice after a 5 h fast with ad libitum access to water. After baseline blood glucose measurement, mice were i.p. injected with 20% glucose (2 g/kg). Changes in blood glucose were measured over 120 min using tail-tip venesection and an Accu-Chek glucometer (Roche). Glucose was administered intraperitoneally rather than orally to bypass the gastrointestinal tract, allowing us to study glucose clearance and insulin secretion independently of glucose-stimulated GLP-1 release and any potentially confounding effects on gastric emptying.

To investigate the effect of indole on acute glucose tolerance, indole (20 mg/kg; I3408; Sigma-Aldrich) or vehicle (water) was orally gavaged 15 min before i.p. injection of glucose. This dose of indole was selected based on a previous study showing an effect of indole on reducing mucosal inflammation [10]. Saline (154 mmol/l NaCl) was used as a vehicle control for all i.p. treatments except FICZ treatment (a synthetic aryl hydrocarbon receptor [AhR] agonist), for which 1% DMSO in saline was used.

Due to the rapid but variable progression of diabetes in TALLYHO mice, there was considerable variability in fasting blood glucose levels across individual mice. The raw IPGTT data were therefore normalised to each mouse’s baseline value (*t*_0_) for meaningful comparisons of glucose excursion.

### Food intake study

Tests were performed in the early light phase on individually caged mice after a 16 h fast. Mice received an oral gavage of indole (20 mg/kg) or vehicle (water), following which pre-weighed amounts of RM1 chow were returned to the cages. Changes in food intake were measured over 2 h.

### Intraperitoneal insulin tolerance test

Insulin tolerance tests were performed on mice after a 5 h fast with ad libitum access to water. After baseline blood glucose measurement, mice were injected with insulin at 1 U/kg (Actrapid, Novo Nordisk). Changes in blood glucose were measured over 120 min using tail-tip venesection and an Accu-Chek glucometer.

For studies in TALLYHO mice, raw blood glucose data were normalised to *t*_0_ to account for variability in diabetes progression.

### Static glucose-stimulated insulin secretion assay

Pancreatic islets were isolated from adult C57Bl6/J mice by collagenase digestion (Universal Biologicals, Cambridge, UK) as previously described [11], and maintained at 37°C, 5% CO_2_ in RPMI medium (Sigma-Aldrich) with 10% FBS (Gibco/Thermo Fisher) and 1% penicillin/streptomycin (Gibco/Thermo Fisher) overnight.

Islets were incubated at 37°C for 1 h in HEPES Krebs Ringer bicarbonate buffer (HKRB) (pH 7.4, bubbled with CO_2_) with 1% BSA and 3 mmol/l glucose (i.e. low glucose). Next, the islets were stimulated with HKRB with 11 mmol/l glucose (i.e. high glucose) in the presence or absence of 1 mmol/l indole. Supernatant samples (which contain secreted insulin) and lysed islet samples were separately collected and stored at −20°C until further use. Insulin levels were assayed using a mouse insulin ELISA kit (Cisbio Bioassays, Codolet, France) according to the manufacturer’s instructions.

### Gut hormone secretion assays

All cell and organoid lines were routinely tested for mycoplasma contamination. STC-1 cells were maintained in DMEM (Gibco/Thermo Fisher) with 10% FBS, 1% penicillin/streptomycin at 37°C, 5% CO_2_. Colonic crypts were isolated from 6–8-week-old C57Bl6/J mice as previously described [12, 13] and plated onto 24-well, 2% Matrigel-coated plates. The crypts were cultured in DMEM (with 10% FBS, 1% penicillin/streptomycin and 10 μmol/l Y-27632 [Bio-Techne, Abingdon, UK]) and incubated overnight at 37°C, 5% CO_2_.

Hormone secretion assays were performed using STC-1 cells and primary murine colonic crypt cultures. The cells were treated using test reagents (made up in DMEM) for 2 h at 37°C, 5% CO_2_. Supernatant and lysed cell samples were collected and stored at −20°C. The levels of GLP-1 and peptide YY (PYY) in cell supernatants and lysates and in plasma were measured using previously described sensitive and specific in-house radioimmunoassays [14, 15].

### Ileal organoid cultures

Ileal organoids were derived from the last 6 cm of the ileum of 6–12-week-old C57Bl6/J and PPG-Cre;GCaMP6 mice, as previously described [16]. Dissociated crypts were resuspended in Cultrex Pathclear reduced growth factor basement membrane extract (Bio-Techne, Abingdon, UK) and cultured in Intesticult medium (STEMCELL Technologies, Cambridge, UK) at 37°C with 5% CO_2_, with medium changes every 2–3 days. Organoids were passaged every 5–7 days at ratios of 1:2–1:3 using Gentle Cell Dissociation Reagent (STEMCELL Technologies, Cambridge, UK) and mechanical disruption.

### Organoid imaging experiments

Organoids from PPG-Cre;GCaMP6 mice were seeded onto 96-well black-walled plates. After 3 days, they were treated with 1 μmol/l 4-hydroxytamoxifen for 24 h to induce GCaMP6f expression in L cells. Timelapse recordings were used to assess calcium response following addition of the treatment. Forskolin (10 μmol/l) and ATP (100 μmol/l) were added after 10 min to confirm cell viability. Imaging was performed using a Nikon ECLIPSE Ti2-E widefield microscope, capturing one frame per second.

Images were analysed using ImageJ with a custom macro package (Intensity_2) from Imperial FILM facility. Regions of interest (ROIs) were drawn around cell bodies to measure fluorescence intensity (*f*). Baseline fluorescence was calculated from 10–30 s prior to treatment addition. Fluorescence change was quantified as Δ*F*/*F*_baseline_, with the maximum value calculated over 10 min post-treatment. Cells were then stratified as responders if the maximum Δ*F*/*F*_baseline_ for the treatment was greater than 20% of *F*_max_ − *F*_min_, and as non-responders if not [17].

### Organoid immunostaining

Murine ileal organoids for imaging were plated onto 8-well chamber slides (Thermo Fisher). After treatment, organoids were fixed using 4% paraformaldehyde, permeabilised using 0.1% Triton-X PBS, and blocked using 5% BSA for 1 h at room temperature. Primary antibody incubation was performed overnight at 4°C using mouse anti-GLP-1 antibody (ab23468, 1:500; Abcam; verified by relative expression, reduced mouse ileal GLP-1 detected when GLP-1 mRNA and circulating protein also reduced). Subsequently, the organoids were washed and incubated with the secondary antibody (anti-mouse Alexa Fluor 647-conjugated IgG, 1:1000; Thermo Fisher) for 1 h at room temperature. DAPI was used as a nuclear stain. Images were acquired using a Leica SP5 confocal laser scanning microscope.

### Tissue processing and immunostaining

Immunohistochemistry was performed according to standard protocols. Tissues were fixed overnight in 4% paraformaldehyde at 4°C, and processed into paraffin blocks. After sectioning using a Leitz 1512 microtome, 5 μm sections were deparaffinised, rehydrated and subjected to antigen retrieval by heating to 90°C in 1 mmol/l EDTA pH 8 solution for 30 min. Next, sections were blocked using 3% BSA in PBS-T (Sigma-Aldrich) for 1 h at room temperature. Primary antibody incubation was performed overnight at 4°C using mouse anti-GLP-1 antibody (ab23468, 1:100; Abcam) and anti-Villin 1 (MA5-16408, 1:100; Thermo Fisher; verified by relative expression, high in Caco-2, COLO 205 and HT-29 cells compared with low or negative in Jurkat and PANC-1 cells and high in mouse jejunum compared with low or negative in mouse colon, kidney and liver). Sections were washed with PBS, and incubated with the secondary antibodies (goat anti-mouse Alexa Fluor 555-conjugated and goat anti-rabbit Alexa Fluor 647-conjugated IgG, 1:300; Thermo Fisher) for 3 h at room temperature. DAPI was used to counterstain the nuclei. Ileum sections were imaged using a Zeiss LSM-780 inverted confocal microscope. The number of GLP-1-immunoreactive cells in stained ileum samples was quantified manually using the ‘Cell Counter’ plug-in in ImageJ, and presented as a proportion (expressed in arbitrary units, AU) of the mean number of GLP-1-positive cells to the mean number of villi counted per field of view. The person counting was blinded to treatment.

### RNA isolation for gene expression analysis by qPCR and sequencing

Organoids were cultured and treated as described in the ‘Ileal organoid cultures’ section above, and snap frozen at −80°C. Total RNA was isolated using TRIsure (Bioline/Meridian Bioscience, Cincinnati, OH, USA) –chloroform extraction after homogenisation with TissueLyser II. After cDNA synthesis using a high-capacity cDNA reverse transcription kit (Applied Biosystems) according to the manufacturer’s instructions, qPCR was performed using Maxima Probe Master Mix (Life Technologies) and Taqman PCR core reagents (Applied Biosystems) in a C1000 CFX384 real-time system thermocycler. Data were analysed using the ${2}^{{\Delta \Delta \mathrm{C}}_{\mathrm{t}}}$ method.

For bulk RNA sequencing, the right liver lobe was dissected from mice and snap frozen at −80°C. Total RNA was purified using the RNeasy Plus Mini kit (Qiagen) according to the manufacturer’s instructions.

### RNA sequencing

Purified total RNA samples were analysed using Nanodrop (*A*_260_/*A*_280_ >1.8, suggesting good RNA purity), and RNA integrity was checked using an Agilent Bioanalyzer RNA 6000 Nano assay (RNA integrity number [RIN] >7). Strand-specific mRNA (polyA) libraries were constructed at Novogene UK, and sequenced using an Illumina NovaSeq 6000 sequencing system with 5.8–7.9 billion bases per sample. Adapters were trimmed using Novogene’s bioinformatics pipeline, and alignments were performed using HISAT2 [18] and mouse genome GRCm38 Ensembl version 102. Uniquely mapped reads were carried forward for gene-level counts and differential gene expression analysis using DESeq2 [19].

### Pathway analysis

The dataset was submitted for Ingenuity Pathway Analysis (http://www.ingenuity.com/). A core analysis was conducted using a cut-off *p* value of <0.05, which reduced the analysis-ready dataset to 716 genes. Stringent filters (Benjamini–Hochberg *p*<0.05, *z* score >2) were applied to detect enriched canonical pathways. For upstream regulator analysis, a *z* score cut-off of >2 and a *p* value for overlap of <0.05 was used.

### Statistical analysis

Data were analysed using GraphPad Prism version 9.0, and are expressed as means ± SD. An unpaired, two tailed Student’s *t* test was used for two-group comparisons. One-way ANOVA with Tukey’s post hoc test was used for single independent variable experiments with more than two groups. Two-way ANOVA with Tukey’s post hoc test was used for analyses with two independent variables. Significance was set at *p*<0.05.

## Results

### Indole improves acute glucose tolerance by potentiating GLP-1 secretion via TRPA1

**Fig. 1:**
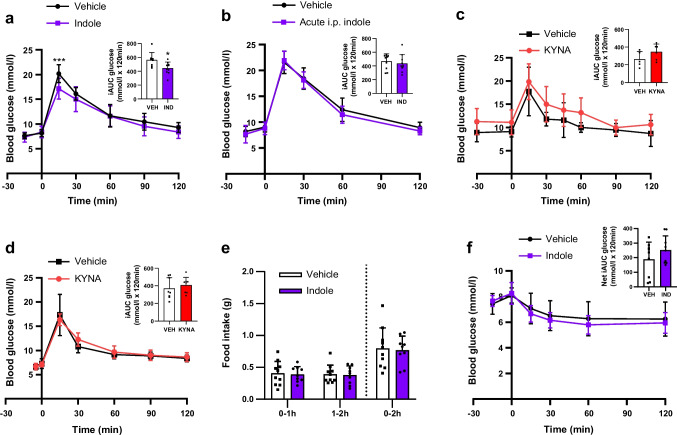
Oral indole improves acute glucose tolerance in mice. (**a**) Blood glucose excursion during an IPGTT after a 5 h fast in lean C57BL6/J mice orally gavaged with indole (20 mg/kg) or water vehicle 15 min prior to glucose load (2 g/kg). *n*=8–10 per group. (**b**) Blood glucose excursion during IPGTT in C57BL6/J mice that received an i.p. injection of either indole (20 mg/kg) or saline vehicle 15 min prior to glucose load. *n*=10 per group. (**c**) Blood glucose excursion during IPGTT in C57BL6/J mice that received an oral gavage of either 10 mg/kg kynurenic acid or water vehicle 30 min prior to glucose load. *n*=7 per group. (**d**) Blood glucose excursion during IPGTT in C57BL6/J mice that received an i.p. injection of either 5 mg/kg kynurenic acid or saline vehicle 5 min prior to glucose load. *n*=8 per group. (**e**) Effect of indole (20 mg/kg) oral gavage or water vehicle on food intake in overnight fasted male mice at the onset of the early light phase for 0–2 h post-administration. *n*=9–10 per group. (**f**) Blood glucose excursion during an IPITT after a 5 h fast in lean C57BL6/J mice that received an oral gavage of 20 mg/kg indole or water vehicle 15 min prior to insulin injection (1 U/kg). *n*=8–9 per group. Insets in (**a**–**d**) and (**f**) present the incremental AUC (iAUC) relative to *t*_0_. Data in the main graphs were analysed by two-way ANOVA with Tukey’s post hoc test. Data in the insets were analysed using Student’s *t* test. Values are means, and error bars represent SD. *p<0.05, ***p<0.001. IND, indole; KYNA, kynurenic acid; VEH, vehicle

An oral indole pre-load significantly improved acute intraperitoneal glucose tolerance (vehicle: 20.4±1.8 mmol/l; indole: 17.1±2.1 mmol/l at *t*_15_; *n*=8–10, *p*<0.001) (Fig. 1a), but an i.p. indole pre-load did not (Fig. 1b), suggesting that the effects of indole are mediated via the gut, potentially by modulating gut hormones. Furthermore, other tryptophan metabolites did not have similar effects on glucose tolerance. Administration of the major tryptophan metabolite kynurenic acid via oral gavage or i.p. injection, at doses that have previously been shown to affect inflammasome activation in colitis [20], did not affect glucose tolerance (Fig. 1c, d). Although previous studies have reported that chronic indole suppresses food intake [21, 22], we did not identify any acute effects of indole on food intake or insulin sensitivity in lean mice (Fig. 1e, f).

**Fig. 2:**
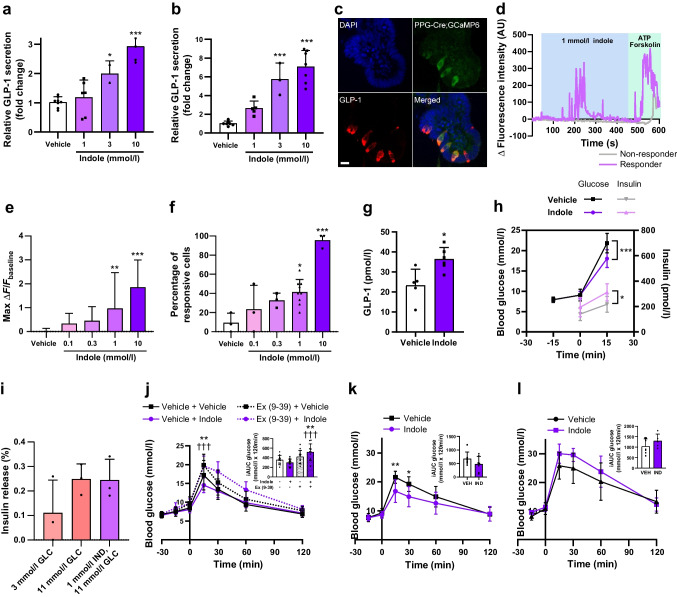
Indole potentiates GLP-1 secretion to improve glucose tolerance. (**a**, **b**) Effect of indole (1–10 mmol/l) on GLP-1 secretion from (**a**) STC-1 cells and (**b**) primary murine colonic crypts, following a 2 h incubation. *n*=3–8 independent plates. (**c**) GLP-1 immunostaining of PPG-Cre;GCaMP6 murine ileal organoids treated with 4-hydroxytamoxifen to induce the expression of GCaMP6 in L cells. Scale bar, 10 μm. (**d**) Representative temporal traces of changes in Ca^2+^ concentration in individual L cells following 1 mmol/l indole treatment of PPG-Cre;GCaMP6 murine ileal organoids, expressed as Δ fluorescence intensity. ATP and forskolin were applied at the end of each imaging session as a positive control. (**e**) Effect of indole (0.1–10 mmol/l) on the magnitude of the L cell calcium response. (**f**) Proportion of L cells showing a calcium response after treatment with 0.1–10 mmol/l indole. *n*=3–12 independent experiments. For each experiment, the percentage was calculated based on 19–90 cells from 7–33 organoids. (**g**) Effect of an oral gavage of indole (20 mg/kg) or water vehicle on plasma GLP-1 concentration 15 min after administration in C57Bl6/J mice following a 5 h fast. *n*=5–6 per group. (**h**) C57Bl/6 mice were pre-treated with an oral gavage of indole (20 mg/kg) or water vehicle 15 min prior to i.p. glucose injection (2 g/kg). Blood glucose levels were measured at *t*_−15_, *t*_0_ and *t*_15_. Plasma insulin levels were measured at *t*_0_ and *t*_15_. *n*=8–9 per group. (**i**) Effect of 1 mmol/l indole in the presence of high glucose (11 mmol/l) on insulin secretion from dispersed murine pancreatic islets, following 1 h static incubation for *n*=3 independent experiments. (**j**) Blood glucose excursion during an IPGTT in C57BL6/J mice that received an i.p. injection of the GLP-1 receptor antagonist exendin (9–39) or saline vehicle 30 min prior to glucose load, and an oral gavage of indole (20 mg/kg) or water vehicle 15 min prior to glucose load. *n*=12–14 per group. (**k**, **l**) Pdx1-CreERT; Glp1r *fl/fl* mice were treated with tamoxifen (100 mg/kg for five consecutive days) to induce knockdown of GLP-1R in Pdx1-expressing beta cells. Blood glucose excursion during IPGTT (**k**) before and (**l**) after tamoxifen treatment. Indole (20 mg/kg) or water vehicle was administered via oral gavage 15 min prior to an i.p. glucose bolus (2 g/kg). *n*=5–6 per group. The insets in (**j**–**l**) present iAUC relative to *t*_0_. Data in (**a**), (**b**), (**e**), (**f**) and (**i**) were analysed by one-way ANOVA with Tukey’s post hoc test. Data in (**h**) and the main graphs in (**j**–**l**) were analysed by two-way ANOVA with Tukey’s post hoc test. Data in (**g**) and the insets for (**j**–**l**) were analysed using Student’s *t* test. Values are means, and error bars represent SD. *p<0.05, **p<0.01, ***p<0.001 for vehicle + vehicle vs vehicle + indole; †††*p*<0.001 for vehicle + indole vs exendin (9–39) + indole. Ex (9–39), exendin (9–39); GLC, glucose; IND, indole; VEH, vehicle

Given previous reports that indole modulates gut hormone secretion in vitro [8, 9], we hypothesised that the acute glucoregulatory effects of indole were mediated via enhanced GLP-1 secretion. We found that increasing concentrations of indole stimulated GLP-1 release from the enteroendocrine STC-1 cell line (10 mmol/l: 2.93±0.43-fold change vs vehicle; *n*=3–8, *p*<0.001) (Fig. 2a) and murine colonic L cell cultures (3 mmol/l: 5.76±1.70; 10 mmol/l: 7.07±1.73-fold change vs vehicle; *n*=3–7, *p*<0.001) (Fig. 2b). Live cell imaging on transgenic organoids generated from PPG-Cre;GCaMP6 murine ileal crypts, in which L cells express the fluorescent Ca^2+^ indicator GCaMP6f (Fig. 2c), showed that indole induced a rapid and reversible dose-dependent elevation of intracellular Ca^2+^, with 10 mmol/l indole increasing the maximum fluorescence intensity by 1.86±1.14-fold (*n*=15–90, *p*<0.001) and also increasing the percentage of responsive L cells (Fig. 2d–f). Correspondingly, oral administration of indole in mice significantly increased plasma total GLP-1 levels after 15 min (vehicle: 23.30±8.10 pmol/l; indole: 36.45±5.73 pmol/l; *n*=5–6, *p*<0.05) (Fig. 2g). Together, these results extend previous in vitro findings showing that indole stimulates GLP-1 release [8, 9]. Furthermore, following oral indole administration in mice, insulin secretion (vehicle: 217.9±64.5 pmol/l; indole: 312.0±67.7 pmol/l at *t*_15_; *n*=7–9, *p*<0.05) increased in parallel with the improvement in glucose tolerance (vehicle: 21.8±2.4 mmol/l; indole: 18.0±2.2 mmol/l at *t*_15_; *n*=7–9, *p*<0.001) (Fig. 2h). However, indole did not potentiate insulin secretion from murine islets in vitro (Fig. 2i), suggesting that it does not directly modulate pancreatic hormone secretion. The acute glucose-lowering effects of oral indole administration were dependent on GLP-1 signalling, as these effects were significantly attenuated by pre-treatment with the GLP-1 receptor antagonist exendin (9–39) (glucose iAUC: vehicle + indole: 299.3±73.82 mmol/l × 120 min; exendin (9–39) + indole: 517.5±170.5 mmol/l × 120 min; *n*=12–14, *p*<0.001) (Fig. 2j). Additionally, there was no longer a beneficial effect of indole on glucose tolerance in Pdx1-CreERT;Glp1r *fl/fl* transgenic mice, in which GLP-1 receptor expression is inducibly knocked down in Pdx1-expressing pancreatic beta cells (Fig. 2k, l). Together, these data suggest that the acute glucoregulatory effects of indole are mediated by increased GLP-1 release driving insulin secretion.

**Fig. 3:**
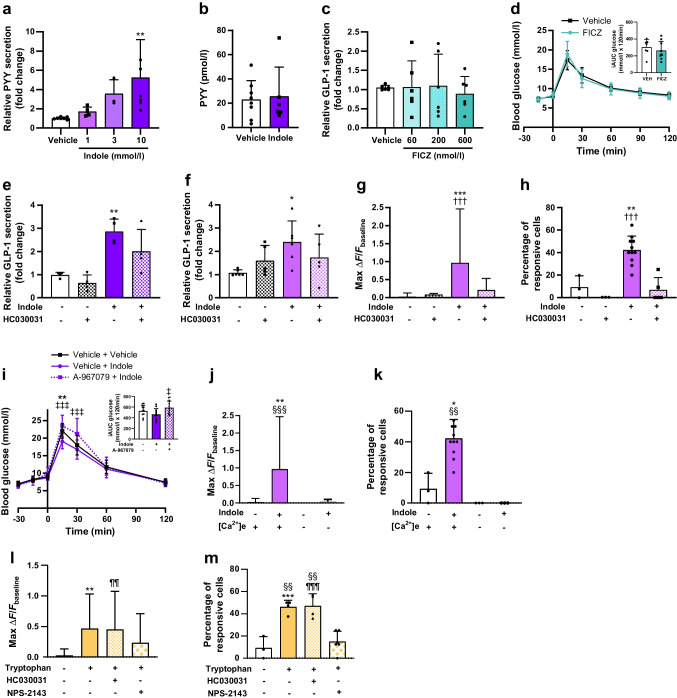
Indole modulates GLP-1 secretion via TRPA1 activity. (**a**) Effect of indole (1–10 mmol/l) on PYY secretion from primary murine colonic crypts, following a 2 h incubation. *n*=3–8 independent plates. (**b**) Effect of an oral gavage of indole (20 mg/kg) or water vehicle on plasma PYY concentrations 15 min after administration in C57Bl6/J mice following a 5 h fast. (**c**) Effect of FICZ (60, 200 or 600 nmol/l) on GLP-1 secretion from STC-1 cells following a 2 h incubation. *n*=6 independent plates. (**d**) Blood glucose excursion during IPGTT in C57BL6/J mice that received an oral gavage of either FICZ (1 µg) or 1% DMSO vehicle 15 min prior to glucose load. *n*=8–10 per group. (**e**, **f**) Effect of indole in the presence or absence of the TRPA1 antagonist HC030031 on GLP-1 release from (**e**) STC-1 cells and (**f**) primary murine colonic crypts following a 2 h incubation. *n*=4–7 independent plates. (**g**) Effects of 1 mmol/l indole in the presence or absence of HC030031 on the L cell calcium response of PPG-Cre;GCaMP6 organoids. (**h**) Proportion of L cells showing calcium mobilisation following 1 mmol/l indole treatment in the presence or absence of 100 μmol/l HC030031; *n*=3–12 independent experiments. For each experiment, the percentage was calculated based on 11–90 cells from 5–33 organoids. (**i**) Blood glucose excursion during an IPGTT in C57BL6/J mice that received an i.p. injection of the TRPA1 inhibitor A-967079 (100 mg/kg) or 6% DMSO, 4% Tween-20 in saline vehicle 30 min prior to glucose load, and an oral gavage of indole (20 mg/kg) or water vehicle 15 min prior to glucose load. *n*=10–12 per group. (**j**) Effects of 1 mmol/l indole in the presence or absence of extracellular Ca^2+^ in imaging buffer on the L cell calcium response of PPG-Cre;GCaMP6 organoids. (**k**) Proportion of L cells showing calcium mobilisation following 1 mmol/l indole treatment in the presence or absence of extracellular Ca^2+^ in imaging buffer; *n*=3–12 independent experiments. For each experiment, the percentage was calculated based on 16–78 cells from 6–30 organoids. (**l**) Effects of 30 mmol/l tryptophan in the presence or absence of 100 μmol/l HC030031 or NPS2143 on the L cell calcium response of PPG-Cre;GCaMP6 organoids. *n*=25–39 cells from 8–13 organoids. (**m**) Proportion of L cells showing calcium mobilisation following 30 mmol/l tryptophan treatment in the presence or absence of 100 μmol/l HC030031 or NPS2143. The insets in (**d**) and (**i**) represent iAUC relative to *t*_0_. Data in (**a**), (**d**–**h**) and (**j**–**m**) were analysed by one-way ANOVA with Tukey’s post hoc test. Data in the main graphs in (**d**) and (**i**) were analysed by two-way ANOVA with Tukey’s post hoc test. Data in (**b**) and the insets for (**d**) and (**i**) were analysed by Student’s *t* test. Values are means, and error bars represent SD. *p<0.05, **p<0.01, ***p<0.001 vs vehicle in (**a**), vs vehicle without indole or HC030031 in (**e–h**), vehicle + vehicle vs vehicle + indole in (**i**), vs vehicle without indole but with [Ca^2+^]_e_ in (**j**, **k**), and vs vehicle without tryptophan, HC030031 or NPS-2143 in (**I**, **m**); ^†††^*p*<0.001 vs HC030031 + indole; ^‡^* p*<0.05, ^‡‡‡^*p*<0.001 for vehicle + indole vs A-967079 + indole; ^§§^*p*<0.01_,_^§§§^*p*<0.001 vs indole + no [Ca^2+^]_e_ in (**j**, **k**) and vs tryptophan + NPS-2143 in (**m**); ^¶¶^*p*<0.01, ^¶¶¶^*p*<0.001 vs vehicle without tryptophan, HC030031 or NPS-2143. [Ca^2+^]_e_, extracellular Ca^2+^

Although 10 mmol/l indole increased the release of PYY, which is co-expressed with GLP-1 in L cells [23], from murine colonic crypt cultures by 5.26±3.94 fold (*n*=7, *p*<0.01) (Fig. 3a), plasma PYY levels were unchanged 15 min after oral indole administration in mice (Fig. 3b). This is in accordance with the lack of effect of indole on food intake in vivo (Fig. 1e), and suggests that any effect on PYY release in vivo was probably short-lived. It may also reflect the relatively low density of PYY-expressing cells in the proximal small intestine.

Indole and its derivatives can modulate intestinal homeostasis and immune functions via the AhR [5], while impaired AhR ligand production has been associated with an increased risk of developing metabolic syndrome [24]. Given that many AhR antagonists have partial agonist actions, and exhibit different efficacies depending on the specific ligand being blocked and the tissue that the receptor is expressed in, we investigated whether AhR agonism plays a role in glucoregulation. However, at concentrations and doses previously shown to stimulate hormone secretion and reduce obesity-induced hyperglycaemia [24], the synthetic AhR agonist FICZ did not stimulate GLP-1 secretion from STC-1 cells (Fig. 3c), nor did it modify glucose tolerance in C57Bl6/J mice (Fig. 3d). This suggests that the AhR is not involved in the acute effects of indole on glucoregulation and GLP-1 secretion, perhaps unsurprisingly given that indole is a relatively weak agonist of the mouse AhR [25].

The excitatory calcium-permeable cation channel transient receptor potential channel subtype A1 (TRPA1) is widely expressed in enteroendocrine cells (EECs), and has been implicated in gut microbial EEC activation in zebrafish, as well as in indole-mediated serotonin secretion from human and murine primary intestinal cultures [9]. TRPA1 activation has also previously been linked to GLP-1 secretion from murine L cells in vitro [26]. We found that indole-induced GLP-1 release from STC-1 cells (Fig. 3e) and murine primary colonic crypt cultures (Fig. 3f) was attenuated in the presence of the TRPA1 inhibitor HC030031, while pre-treatment of PPG-Cre;GCaMP6 organoids with HC030031 blocked indole-stimulated L cell activation (Fig. 3g, h). Additionally, TRPA1 inhibition in mice using the TRPA1 inhibitor A-967079 also blocked indole-mediated improvements in glucose tolerance (glucose iAUC: vehicle + indole: 462.0±108.2 mmol/l × 120 min; A-967079 + indole: 586.9±126.3 mmol/l × 120 min; *n*=12, *p*<0.05) (Fig. 3i). When PPG-Cre;GCaMP6 organoids were imaged in Ca^2+^-free buffer, indole-stimulated Ca^2+^ mobilisation was blocked, reflecting the need for extracellular Ca^2+^ influx for indole to activate L cells (Fig. 3j, k), in accordance with the action of TRPA1.

The indole precursor molecule l-tryptophan can also stimulate GLP-1 release, an effect that is thought to be mediated in part via the Ca^2+^-sensing receptor [27]. While l-tryptophan-induced Ca^2+^ mobilisation in L cells was expectedly attenuated by the Ca^2+^-sensing receptor antagonist NPS-2143, it was unmodified by the TRPA1 inhibitor HC030031 (Fig. 3l, m). Together, these data suggest that the TRPA1 ion channel mediates indole-stimulated GLP-1 secretion and glucose management via a process distinct from that which mediates tryptophan-induced GLP-1 secretion.

### A single bolus of indole improves long-term glucose tolerance by augmenting enteroendocrine differentiation and hence L cell numbers

**Fig. 4:**
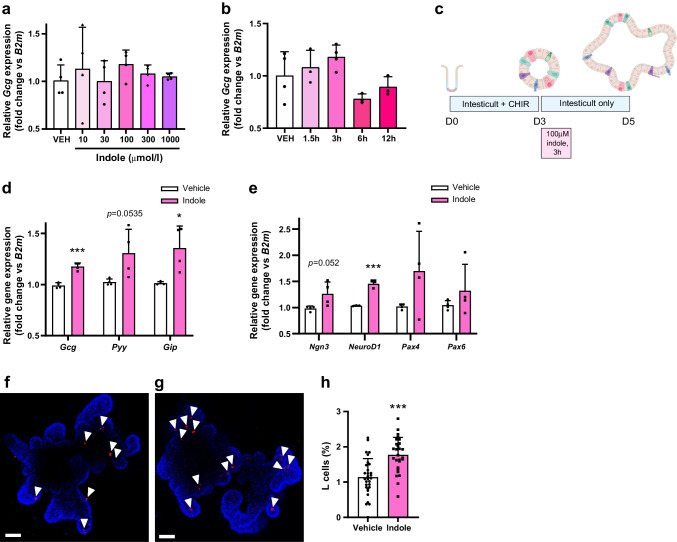
Indole modulates enteroendocrine differentiation to increase L cell abundance in intestinal organoids. (**a**) Effect of 3 h indole (10–1000 μmol/l) treatment on expression of *Gcg*, which encodes the gut hormone GLP-1, in murine intestinal organoids. (**b**) Effect of 100 μmol/l indole treatment for 1.5–12 h on expression of *Gcg* in murine intestinal organoids. (**c**) Experimental paradigm for treatment of WT murine ileal organoids. Organoids were incubated with 100 μmol/l indole or vehicle control for 3 h and collected 2 days later. Created with Biorender.com. (**d**) Expression of the gut hormone genes *Gcg*, *Pyy* and *Gip* in murine intestinal organoids cultured with indole or vehicle. *n*=4 independent experiments. (**e**) Expression of the genes *Ngn3*, *NeuroD1*, *Pax4* and *Pax6*, encoding transcription factors directing L cell development, in murine intestinal organoids cultured with indole or vehicle. *n*=4 independent experiments. (**f**, **g**) Representative images of GLP-1 immunostaining (red) in organoids treated with (**f**) vehicle control or (**g**) 100 μmol/l indole. Nuclei are labelled using DAPI (blue). White arrowheads indicate individual GLP-1 immunoreactive L cells. Scale bar, 50 µm. (**h**) L cell numbers in control organoids and organoids treated with indole. *n*=27–30 organoids. Data in (**a**) and (**b**) were analysed by one-way ANOVA with Tukey’s post hoc test. Data in (**d**), (**e**) and (**h**) were analysed using Student’s *t* test. Values are means, and error bars represent SD. *p<0.05, ***p<0.001 vs vehicle; borderline significant *p* values shown are vs vehicle. CHIR, GSK-3 inhibitor CHIR99021

Specific GLP-1 secretagogues can enhance L cell differentiation [28]. Furthermore, the tryptophan derivative indole-3-acetate has also been shown to modify intestinal lineage differentiation [29]. Having established the acute effects of indole on glucoregulation and GLP-1 secretion, we investigated whether indole mediated longer-term effects on the GLP-1 system. Wild-type (WT) murine ileal organoids were exposed to a 3 h treatment pulse of various concentrations of indole (Fig. 4a) and multiple treatment lengths for 100 µmol/l indole (Fig. 4b), and the effect on expression of *Gcg*, which encodes GLP-1, was determined 2 days later. We subsequently repeated the 3 h treatment pulse study using only the most effective concentration of indole, 100 µmol/l (Fig. 4c). We found a 1.24±0.36-fold (*n*=7, *p*<0.01) upregulation in mRNA expression of *Gcg* in indole-treated organoids, in addition to elevated transcript levels for *Pyy* and *Gip* (Fig. 4d). Indole treatment also increased mRNA expression of the transcription factors *NeuroD1* and *Neurog3* (also known as *Ngn3*), which are associated with L cell endocrine specification (Fig. 4e), indicating enhanced development of enteroendocrine secretory progenitors. Expression of the enteroendocrine differentiation factor genes *Pax4* and *Pax6* was also increased but not significantly (Fig. 4e). In line with the upregulated *Gcg* mRNA expression, addition of 100 µmol/l indole to organoid culture medium for 3 h increased L cell number 1.55-fold (vehicle: 1.14±0.53%; indole: 1.78±0.49%; *n*=27–30, *p*<0.001) (Fig. 4f–h). Thus, a short indole treatment exposure can augment the L cell population in mouse intestinal organoids.

**Fig. 5:**
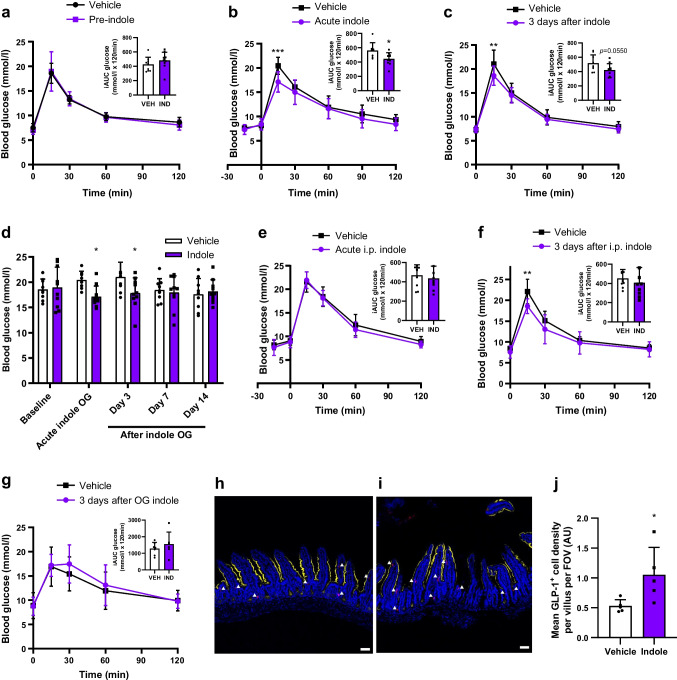
Indole increases L cell abundance to improve long-term glucose tolerance in vivo. (**a**) Blood glucose excursion during an IPGTT in C57BL6/J mice at baseline (prior to treatment intervention). *n*=8–10 per group. (**b**) Blood glucose excursion during an IPGTT in C57BL6/J mice orally gavaged with 20 mg/kg indole or water vehicle 15 min prior to glucose load (2 g/kg). *n*=8–10 per group. (**c**) Blood glucose excursion during an IPGTT in C57BL6/J mice 3 days after a single oral bolus of indole. *n*=8–10 per group. (**d**) Blood glucose levels 15 min after an i.p. glucose bolus (2 g/kg), measured prior to indole administration, on the day of indole administration, and 3, 7 and 14 days after indole administration. *n*=8–10 per group. (**e**) Blood glucose excursion during an IPGTT in C57BL6/J mice that received an i.p. injection of 20 mg/kg indole or saline vehicle 15 min prior to glucose load (2 g/kg). *n*=8–10 per group. (**f**) Blood glucose excursion during an IPGTT in C57BL6/J mice 3 days after a single i.p. bolus of indole. (**g**) Blood glucose excursion during an IPGTT in Pdx1-CreERT;Glp1r *fl/fl* mice 3 days after a single oral indole administration. *n*=6–7 per group. (**h**, **i**) Mice were orally gavaged with 20 mg/kg indole or water vehicle and culled 3 days later. Representative images of ileal sections from (**h**) vehicle-treated and (**i**) indole-treated mice following GLP-1 immunostaining (red). Epithelial cells were stained using anti-Villin 1 (yellow). Nuclei are labelled using DAPI (blue). White arrowheads indicate individual GLP-1 immunoreactive L cells. Scale bar, 50 µm. (**j**) Mean density of GLP-1 immunoreactive L cells in the ileum of vehicle- and indole-treated mice. *n*=5 per group. The insets in (**a**–**c**) and (**e**–**g**) represent iAUC relative to *t*_0_. Data in the main graphs in (**a**–**c**) and (**e**–**g**) were analysed by two-way ANOVA with Tukey’s post hoc test. Data in the insets to (**a**–**c**) and (**e**–**g**) and in (**j**) were analysed using Student’s *t* test. Data in (**d**) were analysed by Student’s *t* test corrected for multiple comparisons. Values are means, and error bars represent SD. *p<0.05, **p<0.01, ***p<0.001 vs vehicle. FOV, field of view; IND, indole; OG, oral gavage; VEH, vehicle

We next investigated whether the effects of indole on epithelial cell fate translated into metabolic benefits in vivo. Prior to a single oral bolus of indole treatment intervention, both groups of C57Bl6/J mice were similarly glucose-tolerant (Fig. 5a). As expected, mice showed improved glucose tolerance after acute oral indole administration (Fig. 5b). This improved glucose tolerance was sustained, with a reduced peak in blood glucose levels following an i.p. bolus of glucose 3 days after oral indole administration (Fig. 5c), despite the half-life of indole previously being reported to be less than 8 h [21]. This effect of indole on glucose tolerance was lost by 7 days post-administration (Fig. 5d). Next, we observed that, while i.p. indole administration (which does not affect GLP-1 secretion) did not modify acute glucoregulation (Fig. 5e), indole-treated mice displayed improved glucose tolerance 3 days post i.p. administration (Fig. 5f). These data suggest that the effect of indole on long-term glucose tolerance is mediated via a mechanism that is distinct from that mediating the acute stimulation of GLP-1 release. However, in Pdx1-CreERT;Glp1r *fl/fl* mice, these effects on glucose tolerance 3 days after oral indole administration were lost (Fig. 5g), suggesting that the mechanism for these longer effects on glucose tolerance is also dependent on GLP-1 receptor signalling. These findings suggest that indole improves acute and long-term glucose tolerance via two distinct mechanisms, but that both involve GLP-1 signalling in pancreatic beta cells.

The intestinal epithelium undergoes rapid renewal, regenerating every 3–5 days. Given that the improvement in glucose tolerance observed in indole-treated mice was lost after approximately a week, and in line with the in vitro findings, we hypothesised that a single dose of indole would increase differentiation of GLP-1 secreting L cells to improve glycaemic control. Ileum samples collected 3 days later from C57Bl6/J mice that received a single oral indole dose showed an increase in L cell density (vehicle: 0.53±0.11 AU; indole: 1.05±0.46 AU; *n*=5, *p*<0.05) (Fig. 5h–j).

Taken together, these data suggest that indole modulates EEC fate specification and increases L cell abundance, improving glucose homeostasis in mice. These findings position indole as a potential glucose-lowering therapy.

### Sub-chronic indole administration improves glucose tolerance and insulin sensitivity in WT and diabetic mice

**Fig. 6:**
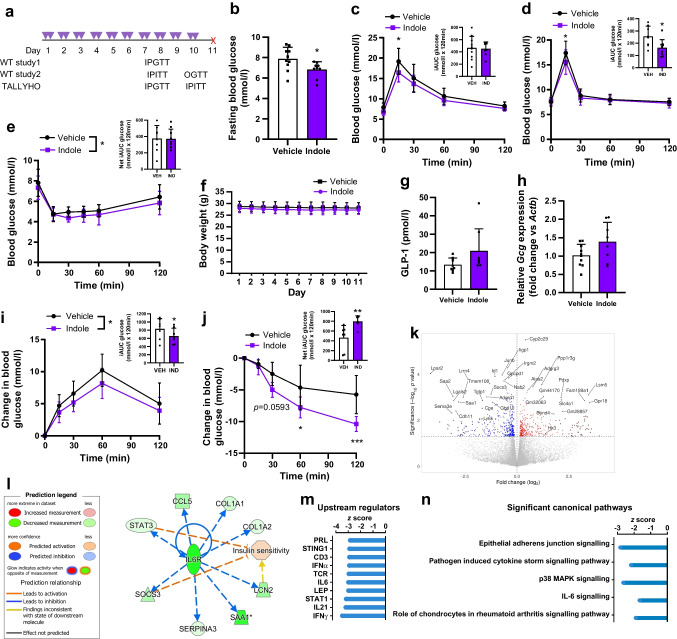
Sub-chronic indole administration improves glucose tolerance and insulin sensitivity in WT mice and a type 2 diabetes mouse model, potentially via inhibition of IL-6 signalling. (**a**) Sub-chronic indole administration study design. Mice received an oral gavage of 20 mg/kg indole or vehicle control (water) twice daily for seven consecutive days. On day 8, an IPITT or IPGTT was performed. The next day, mice received two doses of indole or vehicle. On day 10, an OGTT or IPITT was performed. One day after the second metabolic test, the mice were culled for tissue collection. Created with Biorender.com. (**b**) Fasting blood glucose levels in WT C57BL6/J mice after 7 days of indole or vehicle administration. *n*=9–10 per group. (**c**) Blood glucose excursion during an IPGTT in vehicle- and indole- treated WT C57BL6/J mice. *n*=9–10 animals per group. (**d**) Blood glucose excursion during an OGTT (3 g/kg glucose) after a 5 h fast in vehicle- and indole- treated WT C57BL6/J mice. *n*=8–9 per group. (**e**) Blood glucose excursion during an IPITT (1 U/kg insulin) after a 5 h fast in vehicle- and indole- treated WT C57BL6/J mice. *n*=8–9 per group. (**f**) Body weight of vehicle- and indole- treated WT C57BL6/J mice over the course of the study. *n*=9–10 per group. (**g**) Plasma GLP-1 levels for vehicle- and indole- treated WT C57BL6/J mice. *n*=8–9 per group. (**h**) Relative *Gcg* expression in the ileum of vehicle- and indole- treated WT C57BL6/J mice. *n*=8–10 per group. (**i**) Change in blood glucose excursion (normalised to *t*_0_) during an IPGTT after an overnight fast in vehicle- and indole-treated TALLYHO mice. *n*=7 per group. (**j**) Change in blood glucose excursion (normalised to *t*_0_) during an IPITT in vehicle- and indole-treated TALLYHO mice. *n*=7 per group. Blood glucose in TALLYHO mice was normalised to account for variability in baseline measurements due to differences in diabetes progression. (**k**) Volcano plot of differentially expressed genes between vehicle and indole-treated liver tissues of TALLYHO mice. *n*=7 per group. (**l**) Regulatory network for the IL-6 receptor overlayed with predicted effect on the Ingenuity Pathway Analysis functional gene set for insulin sensitivity. Vertical oval, transmembrane receptor; horizontal oval, transcriptional regulator; triangle, phosphatase; trapezium, transporter; square, cytokine; octagon, function; circle, other. The asterisk (*) after SAA1 indicates a duplicate identifier mapping. (**m**) Top ten inhibited upstream regulators. (**n**) *z* scores for significantly affected (*p*<0.05) canonical pathways derived via Ingenuity Pathway Analysis (gene ontology analysis). The insets in (**c**–**e**), (**i**) and (**j**) represent iAUC relative to *t*_0_. Data in the main graphs in (**c**–**e**), (**i**) and (**j**) were analysed by two-way ANOVA with Tukey’s post hoc test. Data in (**b**), (**g**) and (**h**) and in the insets to (**c**–**e**), (**i**) and (**j**) were analysed using Student’s *t* test. Values are means, and error bars represent SEM. *p<0.05, **p<0.01, ***p<0.001. IND, indole; VEH, vehicle

To further investigate the therapeutic utility of indole in type 2 diabetes treatment, we next studied the effects of sub-chronic indole administration in WT and a type 2 diabetes mouse model (Fig. 6a). WT C57Bl6/J mice that received sub-chronic indole administration showed reduced fasting blood glucose levels (vehicle: 7.9±1.1 mmol/l; indole: 6.8±0.7 mmol/l; *n*=5, *p*<0.05) (Fig. 6b), in addition to improvements in intraperitoneal glucose tolerance (vehicle: 19.2±3.2 mmol/l; indole: 16.4±2.3 mmol/l at *t*_15_; *n*=8–10, *p*<0.05) and oral glucose tolerance (Fig. 6c, d); a modest increase in insulin sensitivity was also noted (Fig. 6e). Furthermore, these effects were independent of any changes in body weight (Fig. 6f). Mice given sub-chronic indole treatment also demonstrated a trend towards elevated basal circulating GLP-1 levels and higher *Gcg* expression in the ileum, although these changes did not reach statistical significance (Fig. 6g, h). These data are in line with findings that chronic indole supplementation in mice reduced high-fat diet-associated systemic insulin resistance and glucose intolerance, although this is the first study to report metabolic improvements after a relatively short treatment intervention and in WT mice. Given these metabolic benefits of indole, and the preservation of GLP-1’s insulinotropic effect in type 2 diabetes, the same sub-chronic indole treatment protocol was repeated in TALLYHO/JnG mice, a polygenic type 2 diabetes mouse model that exhibits hyperglycaemia and insulin resistance, reflecting the multifactorial nature of human type 2 diabetes [30]. Indole-treated TALLYHO/JnG mice showed improved glucose tolerance (Fig. 6i), and, interestingly, an improvement in insulin sensitivity (net glucose iAUC: vehicle 464.2±249.2 mmol/l × 120 min; indole 795.6±114.6 mmol/l × 120 min; *n*=7, *p*<0.01) (Fig. 6j), demonstrating the potential translatability of indole’s metabolic effects to a disease state. To further investigate this insulin sensitivity phenotype, we profiled whole-liver transcriptomes from indole- and vehicle-treated TALLYHO mice. A total of 719 differentially expressed genes (unadjusted *p*<0.05) were found (Fig. 6k), including upregulation of *Hk3*, which encodes the rate-limiting enzyme of many glucose metabolism pathways, representing a possible mechanism by which indole improves hepatic insulin sensitivity. Ingenuity Pathway Analysis further revealed that indole induces mild transcriptional alterations in liver metabolism; specifically, an inhibition of IL-6 cytokine signalling (Fig. 6l–n). The anti-inflammatory effects of indole in the liver could potentially also enhance insulin sensitivity, consistent with work showing that indole and related compounds exert hepatoprotective effects in metabolic dysfunction-associated steatotic liver disease (previously known as non-alcoholic fatty liver disease) by reducing the production of proinflammatory mediators [21, 31, 32]. Furthermore, modulation of cell–cell and tight-junction assembly pathways was observed, in line with previous reports of indole’s role in maintaining epithelial barrier function (Fig. 6n) [6, 7].

## Discussion

We have shown that the bacterial tryptophan metabolite indole can act on the gastrointestinal tract to modify GLP-1 secretion from enteroendocrine L cells using several models. The acute stimulatory effect appears to involve the TRPA1 ion channel, but not the AhR, potentially by modulating extracellular Ca^2+^ flux. However, as TRPA1 inhibition of indole-induced GLP-1 release was incomplete in STC-1 cells and colonic crypt cultures, it is possible that other Ca^2^⁺-dependent pathways also contribute to GLP-1 release, highlighting the complexity of the underlying mechanisms. Importantly, we also demonstrate that the enhanced GLP-1 secretion and hence insulin secretion in response to indole translate into improved acute glucoregulation in vivo. Interestingly, indole seemed to acutely worsen glucose tolerance in vivo when GLP-1R signalling was blocked, suggesting that the beneficial effects of indole are primarily GLP-1-dependent and may mask underlying harmful effects.

A previous study identified time-dependent, biphasic effects of indole on GLP-1 release, with suppression observed following prolonged exposure [8]. Our studies partly align with these findings: short-term exposure triggers rapid Ca^2^⁺ responses, but our secretion assays primarily used a 2 h incubation window, which partly overlaps with the ‘prolonged exposure’ in the previous study. Nonetheless, we note that treatment periods of longer than 2 h or high-dose indole treatment reduced *Gcg* mRNA expression in ileal organoids, suggesting that transient, pulse-like exposure to specific concentrations of indole may more effectively promote glucose management than sustained administration of high doses. Differences in the models and gut regions investigated may explain the different timelines for downregulation in our study and the previous study [8], highlighting the need for more comprehensive time course studies across various in vitro models and in vivo.

Furthermore, our work represents the first organoid study to investigate the effects of indole in the modification of EEC specification. Interestingly, indole can increase neurogenesis in the adult mouse hippocampus [29], and the transcriptional pathways implicated in gut endocrine specification and neuronal differentiation share some commonalities [33, 34]. Nonetheless, future studies are required to elucidate indole’s effects on various intestinal cell types, as well as its transcriptional targets and regulatory pathways. Remarkably, we also found that the glucose-lowering benefits conferred by indole persisted for several days following administration, most likely due to an increase in L cell abundance. Although we were unable to directly quantify GLP-1 secretion at later timepoints, it would be helpful to determine whether the glucose tolerance improves or wanes concurrently with increases and decreases in L cell density and circulating GLP-1 levels, and whether more extended treatment with indole promotes longer-term changes in EEC populations.

Interestingly, sub-chronic indole treatment improved insulin sensitivity in both healthy mice and diabetic mice models, with the most pronounced effect being observed in the TALLYHO/JnG model of type 2 diabetes. In this diabetic context, transcriptomic analysis of liver tissue suggested that indole’s actions may involve suppression of inflammatory pathways, aligning with its recognised anti-inflammatory properties [21, 31, 32]. However, as transcriptomic changes could be secondary to enhanced insulin sensitivity, the exact contribution of these pathways to indole’s metabolic benefits warrants further investigation. Although there was a trend for indole to elevate *Gcg* expression and basal GLP-1 levels in healthy mice, these changes were not statistically significant, and additional work is required to determine whether sub-chronic indole administration promotes L cell development or sustained hormone release.

In conclusion, our work has identified indole as a novel signalling molecule for the potential treatment of diabetes, providing further insight into its role in glucose management and L cell differentiation. Further work is required to determine whether this pathway is conserved in humans, but our findings raise the novel possibility of incorporating indole into nutraceutical supplements to treat and/or prevent glucose intolerance associated with type 2 diabetes.

## Data Availability

All data, methods and unique/stable reagents generated in this study will be made available from the corresponding author upon reasonable request, but may require a completed Materials Transfer Agreement. RNA sequencing data are available from the Gene Expression Omnibus under accession number GSE306720.

## Abbreviations

AhR: Aryl hydrocarbon receptor
AU: Arbitrary units
EEC: Enteroendocrine cell
GLP-1: Glucagon-like peptide-1
GLP-1R: Glucagon-like peptide-1 receptor
PYY: Peptide YY
TRPA1: Transient receptor potential channel subtype A1
WT: Wild-type
